# Correction: GPR18 Agonist Resolvin D2 Reduces Early Brain Injury in a Rat Model of Subarachnoid Hemorrhage by Multiple Protective Mechanisms

**DOI:** 10.1007/s10571-024-01475-4

**Published:** 2024-04-24

**Authors:** Tongyu Zhang, Gang Zuo, Hongqi Zhang

**Affiliations:** 1 Department of Neurosurgery, Xuanwu Hospital, Capital Medical University, 45 Changchun St., Beijing, 100053 China; 2 Department of Neurosurgery, The Affiliated Taicang Hospital, Soochow University, Taicang, Suzhou, 215400 Jiangsu China

**Correction: Cellular and Molecular Neurobiology (2022) 42:2379–2392** 10.1007/s10571-021-01114-2

The original version of this article unfortunately contained error in Figs. 8 and 9.

In Fig. 8, the two DAPI images in Fig. 8b should be reversed.

In Fig. 9, the author mistakenly used the wrong image and so the error occurred in protein band (Cleaved Caspase-3) and the corresponding statistical result.

Also, the authors confirm that these corrections do not affect the experimental results or conclusions.

The correct Figs 8 and 9 are given here.

**Fig. 8:**
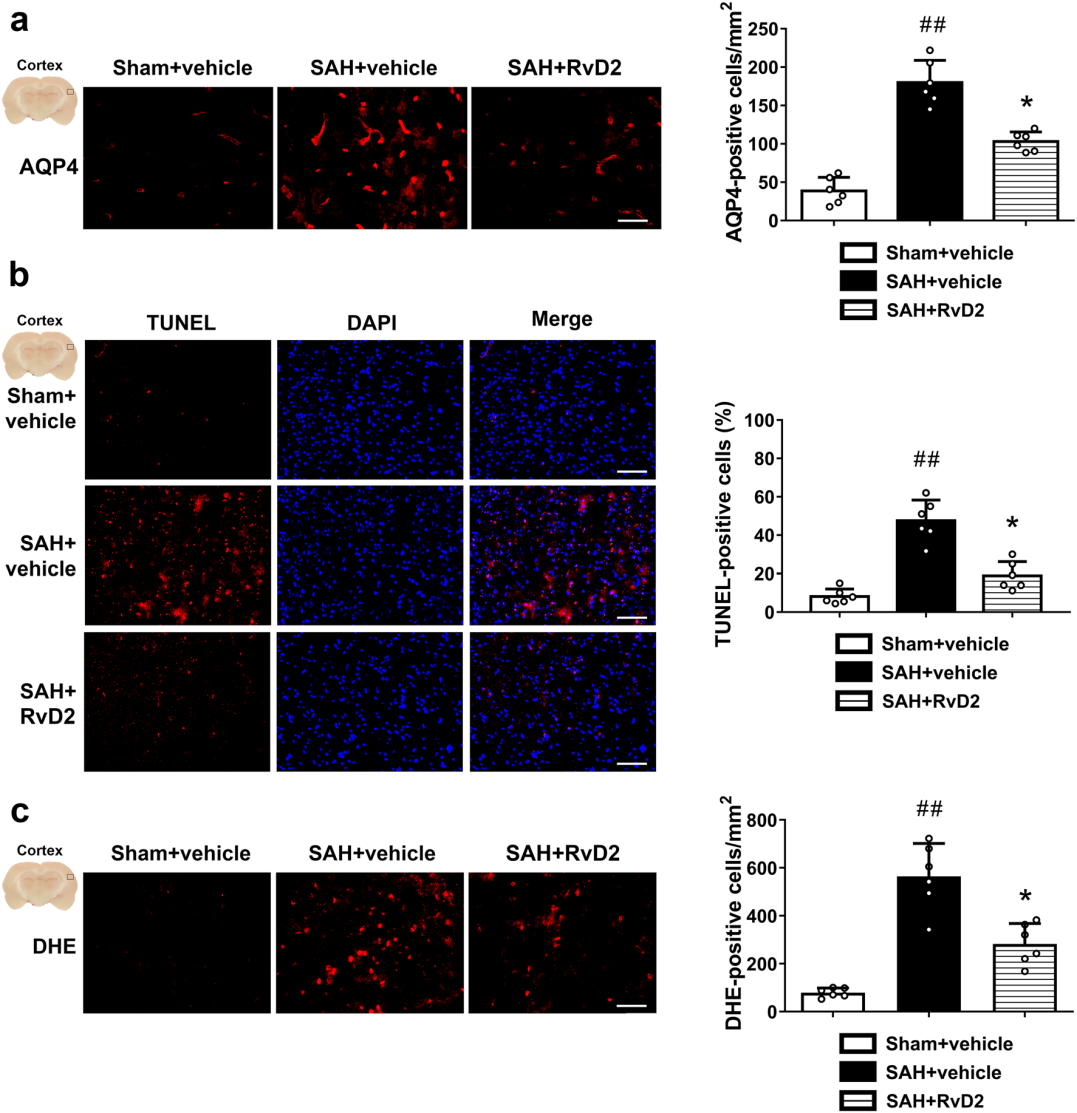
Immunostaining studies to show that RvD2 protected BBB and attenuated apoptosis and oxidative stress in cortex after SAH. **a** Representative immunostaining for AQP4, and quantitative analysis of AQP4-positive cells. **b** Representative TUNEL staining, and quantitative analysis of apoptotic index (percentage of TUNEL-positive cells). **c** Representative DHE staining, and quantitative analysis. Scale bars = 200 μm. Bars represent the mean ± SD (n = 6 from each group). ^##^*P* < 0.01 vs. Sham + vehicle group; *P < 0.05 vs. SAH + vehicle group

**Fig. 9:**
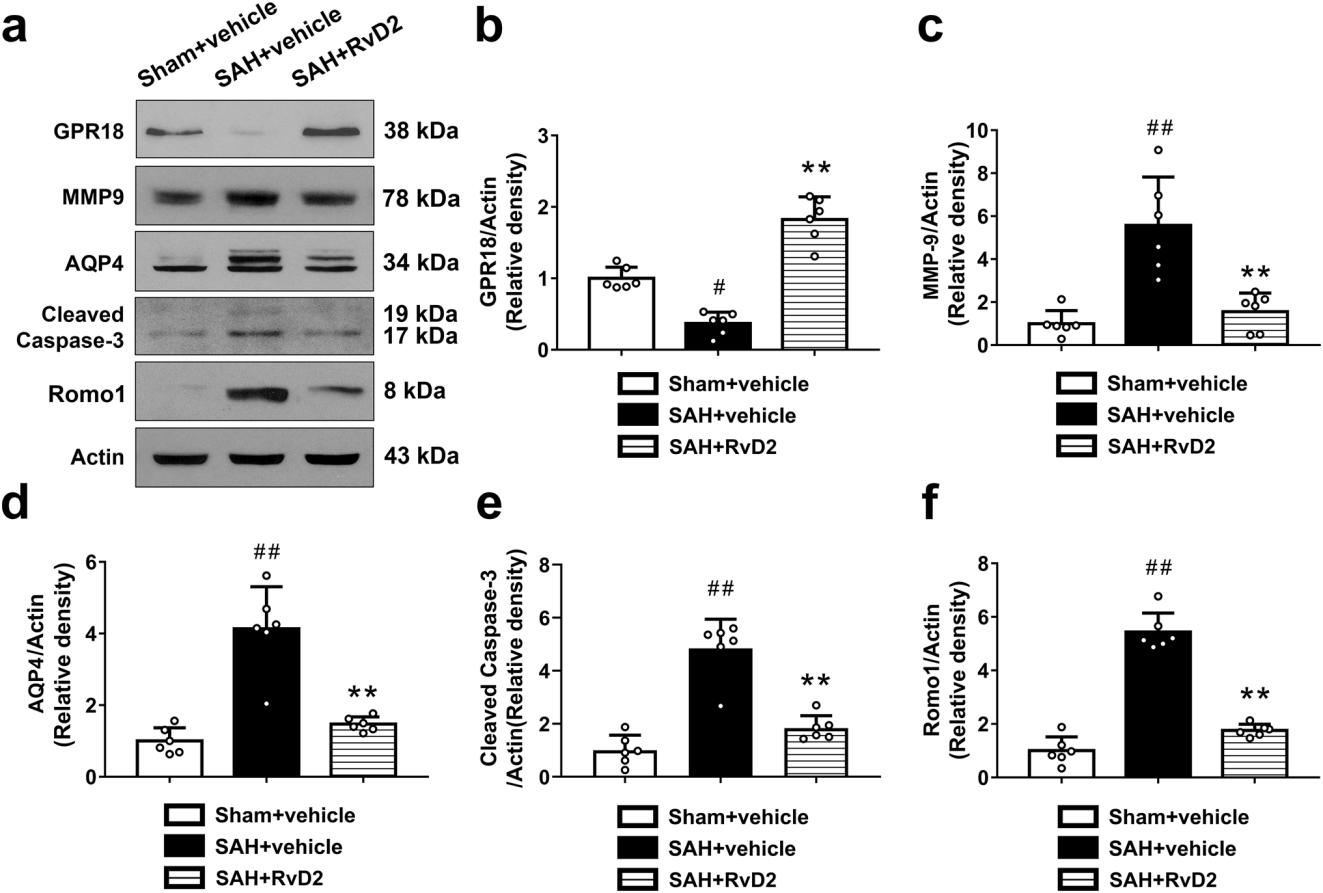
Western blotting studies to show that RvD2 protected BBB as well as attenuated apoptosis and oxidative stress in cortex after SAH. **a** Representative Western blots of GPR18, MMP-9, AQP4, cleaved caspase-3, and Romo1 in cortex. **b–f** Quantitative analysis of the Western blots for each protein. Bars represent mean ± SD (n = 6 from each group). _#_*P*< 0.05, _##_*P* < 0.01. Sham + vehicle group; **P < 0.01 vs. SAH + vehicle group

The original article has been corrected.

